# *Bacillus aerius* synergizes with coal gangue to enhance *Medicago sativa* growth via soil microbiome and gene regulation

**DOI:** 10.1128/aem.00268-26

**Published:** 2026-04-24

**Authors:** Mingwu Liu, Meili Du, Zijie Xi, Kuanysh T. Tastambek, Yuan Bao, Xiaonan Song, Anning Zhou, Yaya Wang

**Affiliations:** 1 College of Chemistry and Chemical Engineering, Xi’an University of Science and Technology, Xi’an, China; 2 Sustainability of Ecology and Bioresources, Al-Farabi Kazakh National University 98799, Almaty, Kazakhstan; 3 Ecology Research Institute, Khoja Akhmet Yassawi International Kazakh-Turkish University 203402 , Turkistan, Kazakhstan; 4 College of Geology and Environment, Xi’an University of Science and Technology, Xi’an, China; Colorado School of Mines, Golden, Colorado, USA

**Keywords:** coal gangue, *Bacillus aerius*, soil improvement, metagenomic analysis

## Abstract

The extensive accumulation of coal gangue poses significant environmental threats through water contamination, soil degradation, and atmospheric pollution, necessitating the urgent development of ecological utilization strategies. This study elucidates the mechanistic basis by which the thermophilic bacterium *Bacillus aerius* (*B. aerius*) enhances plant growth in coal gangue-amended sandy soils. Through integrated analysis of nutrient dynamics, phytohormonal activities, soil enzymatic profiles, and metagenomic functional profiling, we demonstrate significant synergy between coal gangue and *B. aerius*. When applied together in sandy soils, the germination rate, plant height, root length, and fresh biomass of *Medicago sativa* (alfalfa) increased by 1.18–2.06 times. The levels of soil nitrogen, phosphorus, and potassium also significantly increased, resulting in notable improvements in soil fertility. The bacterial treatment enhanced the activities of indole-3-acetic acid, 1-aminocyclopropane-1-carboxylate (ACC) deaminase, and various soil enzyme activities while also optimizing the microbial community structure and increasing the abundance of beneficial bacteria, including *Bacillus*. Metagenomic analysis revealed the upregulation of growth-promoting genes such as *acdS*, *nifK*, and *phnG*, which collectively drive plant growth through multiple pathways, including enhanced soil nutrient availability, hormone regulation, soil enzyme activities, and nutrient cycling. Collectively, this work deciphers molecular-scale bacteria-gangue synergism, providing a theoretical foundation for sustainable coal gangue utilization and ecological restoration of degraded soils.

**IMPORTANCE:**

The accumulation of coal gangue poses significant environmental challenges, necessitating the development of eco-friendly utilization strategies. This study demonstrates that the thermophilic bacterium *Bacillus aerius* acts synergistically with coal gangue to promote alfalfa growth in sandy soils while improving soil fertility. The combined treatment enhanced plant morphological traits, soil nutrient availability, beneficial microbial communities, and associated biological activities, with these effects supported by molecular evidence. As the first study to verify this growth-promoting mechanism, our findings address a critical knowledge gap and provide a theoretical foundation for the sustainable utilization of coal gangue in the ecological restoration of degraded soils.

## INTRODUCTION

Coal gangue, a major solid waste product generated during coal mining and processing, comprises excavation gangue, coal mining gangue, and coal washing gangue (1). Its composition is complex, dominated by silicon dioxide and aluminum oxide but also containing trace amounts of elements such as iron, calcium, magnesium, sodium, potassium, and phosphorus, alongside rare metal oxides (2). The presence of heavy metals raises environmental concerns, as prolonged exposure to water can leach harmful substances into soil and water sources (3). This contamination adversely impacts water quality, ecosystems, agricultural productivity, and groundwater safety (4, 5). Despite these challenges, coal gangue is employed in power generation, cement and concrete production, brick manufacturing, paving materials, and notably, as an agricultural fertilizer (6–8). It is rich in trace elements that can improve soil structure, enhance fertility, and stimulate plant growth, particularly in soils like sandy and saline-alkali types (9). Furthermore, coal gangue serves as a potential substrate for microbial immobilization and can contribute to nitrogen fixation and potassium activation in mineral fertilizer production, ultimately improving soil quality and supporting plant health (10).

*Bacillus aerius* (*B. aerius*) is a naturally ubiquitous bacterium renowned for its ability to form resistant spores, enabling survival under extreme conditions, including drought, high temperatures, and radiation (11, 12). Commonly isolated from air, soil, plant surfaces, and water, it can also disseminate aerially (13). A key characteristic of *B. aerius* is its potent capacity for decomposing organic matter, underpinning its value across agriculture, environmental protection, and industry (12, 14). In agriculture, its thermostable enzymes show promise as biocatalysts for biorefining lignocellulosic biomass. For instance, its ability to saccharify pretreated corn cobs at elevated temperatures could facilitate biofuel production (15). Specific strains, such as *B. aerius* MH1RS1, have been shown to secrete the phytohormone indole-3-acetic acid (IAA), enhance activity of the enzyme 1-aminocyclopropane-1-carboxylate deaminase (ACC deaminase), tolerate salt stress, and directly promote plant growth (16). This salt tolerance, partly mediated by extracellular polysaccharide production, makes such strains suitable candidates for agricultural biotechnology applications (17). Additionally, *B. aerius* effectively degrades recalcitrant waste like chicken feathers, converting them into nutritionally enhanced hydrolysates usable in livestock feed or as biofertilizers (18, 19). Studies also indicate its potential as a biocontrol agent (20), and as a beneficial aquaculture feed supplement, where it enhances growth, disease resistance, and innate immunity in fish species such as *Pangasius bocourti* (21).

Microbial degradation of coal gangue has emerged as a significant research focus for enhancing its utilization (22). While coal gangue is used to produce both organic and mineral fertilizers, microbial treatment offers a promising avenue for improvement (23). For instance, Zhu et al. demonstrated that coal gangue treated with *Stenotrophomonas maltophilia* contained higher levels of phosphorus, potassium, and available silicon than untreated material (24). Similarly, Chen et al. isolated a mixed culture of ferrous iron-oxidizing and sulfur-oxidizing bacteria from coal gangue piles. Bioleaching experiments in a column reactor showed these bacteria enhanced elemental release, achieving a 78.79% desulfurization rate and mitigated the phytotoxic effects of coal gangue (25). Further studies indicate that *Bacillus velezensis* can improve the efficacy of coal gangue as a mineral fertilizer for alfalfa (26), and gangue-based mixed fertilizers have outperformed specialized fertilizers in promoting apple tree trunk growth and new shoot length (27). Significantly, our previous study has shown that *B. aerius* can effectively degrade coal gangue and release beneficial elements (28), positioning microbial degradation as a viable strategy for enhancing coal gangue resource utilization. Despite these advancements, comprehensive understanding remains limited regarding bacterial-induced alterations in key growth-promoting factors and secreted enzyme activities within coal gangue-amended soils. Moreover, the specific mechanisms through which bacteria facilitate plant growth in such environments are poorly elucidated.

This study aims to elucidate the mechanism by which *B. aerius* promotes plant growth in coal gangue-amended sandy soils. We investigated nutrient content changes in soils supplemented with both coal gangue and *B. aerius*. Furthermore, we quantified key plant growth-promoting hormones and enzymatic activities (urease, protease, phosphatase, amylase, and laccase) to assess their contribution to nutrient cycling and plant development. To elucidate the molecular basis of these effects, metagenomic analysis was employed to characterize functional genes and metabolic pathways underlying the observed growth promotion. This study provides mechanistic insights into how *B. aerius* enhances plant growth in coal gangue-amended environments.

## MATERIALS AND METHODS

### Soil matrix and source of bacteria

Coal gangue samples were collected from the Dahaize Coal Mine in Shenmu County, Shaanxi Province, China. Elemental composition and heavy metal content were determined using X-ray fluorescence (XRF) spectrometry, and the results are presented in Tables S1 and S2 at https://doi.org/10.6084/m9.figshare.31852120. Following collection, samples were sealed in sterile polyethylene bags and transported to the laboratory for processing. The bacterial strain employed in this research, *B. aerius* (GenBank accession: MN098858.1), was originally isolated from local soil surrounding the coal gangue deposits. This strain was selected based on its demonstrated coal gangue degradation capability.

### Pot experiments

Plastic pots (15.5 cm diameter, 10.9 cm height) were filled with 500 cm³ of soil matrix. Four experimental treatments were established in triplicate. Sandy Soil Control (C-1): Sandy soil only, amended with an equal volume of sterile culture medium. Coal Gangue Amendment (C-2): Sandy soil mixed with coal gangue. Bacterial Treatment (Y33-1): Sandy soil inoculated with *B. aerius*. Combined Treatment (Y33-2): Sandy soil mixed with coal gangue and inoculated with *B. aerius*. Sixty alfalfa seeds were sown evenly per pot with uniform row spacing. At 7 days post-germination, seedlings were reduced to 20 uniform, healthy plants per pot to eliminate intraspecific competition for space and nutrients. Following a 60-day cultivation period, all plant samples were harvested for subsequent parameter determination. Growth parameters, including germination rate, root length, plant height, and fresh weight, were measured.

### Analysis of soil physicochemical properties

Following plant harvest, soil samples were collected from each pot and stored in pre-labeled sterile polyethylene bags for physicochemical analysis. Key soil parameters were determined according to standard procedures outlined in Soil Agricultural Chemical Analysis (29). These included the following: pH, electrical conductivity (EC), moisture content, total nitrogen (TN), total carbon (TC), carbon-to-nitrogen ratio (C/N), organic matter (OM), total phosphorus (TP), total potassium (TK), and humic-like substances (humic acid, HA; fulvic acid, FA; humin) (30). Soil pH was measured via a calibrated PHS-3C pH meter in a 1:2.5 (wt/vol) soil-to-water suspension. Moisture content was analyzed gravimetrically by oven-drying, then converted to volumetric moisture based on measured bulk density. TN was quantified via the Kjeldahl method. Briefly, dried soil samples (0.1 g) were digested at 500°C for 30 minutes with concentrated H₂SO₄ and a selenium catalyst. The resulting ammonium sulfate [(NH₄)₂SO₄] was distilled, and the liberated NH₃ was trapped in 4% boric acid (adjusted to pH 4.65). Nitrogen concentration was subsequently determined by titrating the boric acid solution. TP was measured using the perchloric acid-sulfuric acid digestion method. TK was quantified following alkali fusion using atomic absorption spectrophotometry. TC and OM content were assessed using the potassium dichromate (K₂Cr₂O₇) oxidation method with external heating. Humic substances (humic acid/HA, fulvic acid/FA, humin) were sequentially extracted and quantified as previously described (Bao, 2000). Briefly, soil samples were mixed with 0.1 mol/L Na₄P₂O₇-NaOH solution at a solid-to-liquid ratio of 1:10 (wt/vol). The mixture was shaken at 180 rpm for 24 hours at 25°C to solubilize humic complexes, followed by centrifugation at 5,000 × *g* for 10 minutes to separate the humic extract from the humin residue. The HA and FA fractions were subsequently isolated via acid precipitation. The carbon content of each fraction was then determined using external-heating potassium dichromate oxidation coupled with volumetric titration.

### Soil plant growth-promoting hormone analysis

The content of IAA was determined following the protocols described previously (31). In brief, the extraction of soil IAA involved suspending soil samples in sterile deionized water at a 1:2.5 soil-to-water ratio. After thorough vortex mixing, 1 mL aliquots of this suspension were centrifuged. An equal volume of Salkowski reagent (S2 colorimetric solution) was then added to 100 µL of the supernatant. The absorbance was subsequently measured at 530 nm using a spectrophotometer. The ACC deaminase activity was measured according to the method reported by others (32).

### Determination of biological enzyme activity in soil

The activity of amylase is determined spectrophotometrically using starch as substrate and dinitrosalicylic acid (DNS) as a chromogenic agent, measuring reducing sugar production at 540 nm (33). Phosphatase activity was determined with disodium phenyl phosphate substrate via absorbance at 400 nm (34). Laccase activity was analyzed by detecting the oxidation of 2,2′-azino-bis (3-ethylbenzothiazoline-6-sulfonic acid) (ABTS), with changes in absorbance recorded at 420 nm (35). Urease activity was evaluated according to the release of ammonium nitrogen (36), and protease activity was determined via a casein hydrolysis assay (37).

### Metagenomic analysis

The Mag-Bind Soil DNA Kit was utilized to extract genomic DNA from all samples, according to the manufacturer’s protocols. DNA libraries were constructed and subjected to paired-end sequencing via the Illumina NovaSeq platform at Majorbio Biopharm Technology Co., Ltd. (Shanghai, China). Adapter sequences and low-quality reads (length <50 bp, quality value <20, or containing N bases) were filtered out using fastp (v0.20.0; https://github.com/OpenGene/fastp). Assembly of metagenomic sequences was carried out with MEGAHIT (v1.1.2; https://github.com/voutcn/megahit) (38). Functional annotation was performed by aligning sequences against the eggNOG database for Clusters of Orthologous Groups (COG) classification and the KEGG database for metabolic pathway annotation.

### Quantitative reverse transcription-PCR

Bacterial RNA was extracted from soil specimens using the OMEGA Soil RNA Mini Kit. RNA integrity was verified by electrophoresis, and purity was assessed spectrophotometrically (A₂₆₀/A₂₈₀ ratio >1.8). Complementary DNA synthesis was performed using 1 μg of total RNA and reverse transcriptase, following the manufacturer’s guidelines. Quantitative PCR (qPCR) amplification was performed in triplicate reactions, each containing cDNA, gene-specific primers (see Table S1 at https://doi.org/10.6084/m9.figshare.31852120), and SYBR Green master mix. Absolute quantification of target gene copies was determined using a plasmid-derived standard curve. The 16S rRNA gene served as the endogenous reference for normalization, with data analyzed via the comparative Cq (ΔΔCq) method.

### Data analysis

All plant growth parameters, soil physicochemical properties, and enzyme activity data were analyzed using SPSS Statistics software. Significant differences among the experimental groups were assessed using one-way analysis of variance, followed by Duncan’s multiple range test. Results were visualized using OriginPro 2018, with graphical representations directly reflecting the statistical significance outcomes.

## RESULTS

### Growth-promoting effects of coal gangue and *B. aerius* on *Medicago sativa*

**Fig 1:**
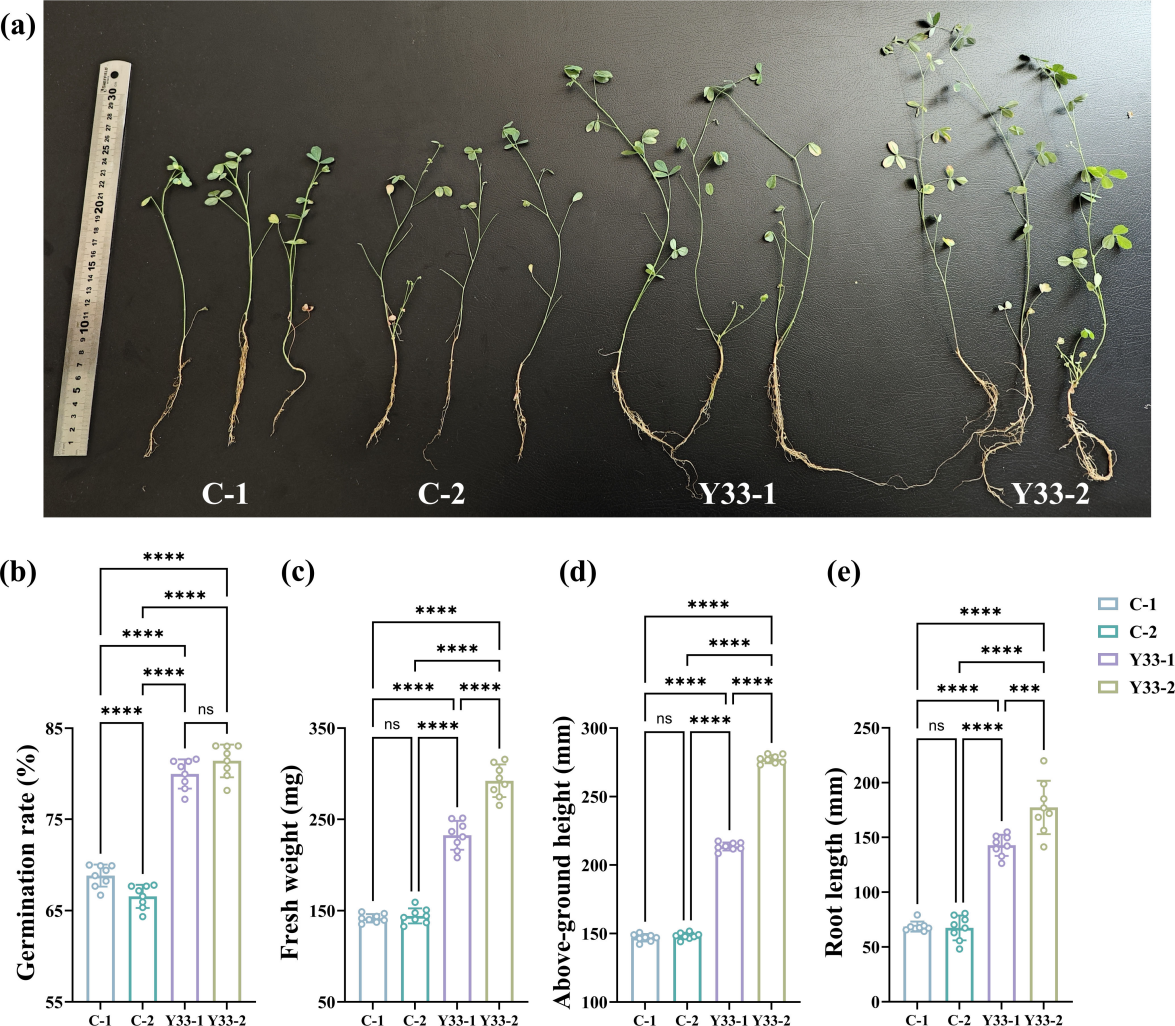
(**a**) Images of alfalfa plants grown under various experimental treatments. The treatments include the following: C-1 (sandy soil), C-2 (sandy soil amended with coal gangue), Y33-1 (sandy soil with *B. aerius* inoculation), and Y33-2 (sandy soil amended with coal gangue and *B. aerius* inoculation). Growth parameters of alfalfa after 60 days of cultivation. The bar graph depicts the following: (**b**) the average germination rate, (**c**) the average fresh weight, (**d**) the average above-ground height, and (**e**) the average root length of alfalfa plants. Statistical significance is indicated as (****, *P* < 0.0001; ***, *P* < 0.001). Error bars represent the mean ± standard deviation (SD) for each replicate experiment.

To evaluate the synergistic effects of coal gangue and *B. aerius* on the growth of *Medicago sativa*, a series of controlled pot experiments was conducted, with each treatment performed in triplicate. Under optimal conditions, the addition of *B. aerius* and coal gangue into the sandy soils (Y33-2 group) resulted in significant improvements in multiple growth parameters of alfalfa (Fig. 1). Specifically, germination rate increased by 1.18-fold (*P* < 0.001), shoot height by 1.91-fold (*P* < 0.001), root length by 2.56-fold (*P* < 0.001), and fresh biomass by 2.06-fold (*P* < 0.001) compared to the untreated control (C-1 group). These results indicate that the combined application of coal gangue and *B. aerius* significantly enhances early growth and biomass accumulation in alfalfa, highlighting its potential for sustainable agriculture and soil remediation.

### Treatment effects on soil physicochemical properties

The impact of coal gangue and *B. aerius* on soil physicochemical properties was assessed across experimental treatments (Table 1). The addition of coal gangue (C-2) significantly reduced soil pH compared to the sandy soil control (C-1), consistent with previous reports on coal gangue’s acidifying potential (39). Additionally, the combined application of coal gangue and *B. aerius* (Y33-2) substantially enhanced soil organic matter content. In the coal gangue-only amendment group (C-2), total soil organic matter (SOM) content increased from 1.41 g/kg in the sandy soil control (C-1) to 31.28 g/kg. This increase was primarily attributable to the direct input of total oxidizable carbon from coal gangue, including residual coal and other inert carbon components. Notably, compared with the C-2 group, the combined treatment of coal gangue and *Bacillus aerius* (Y33-2) exhibited further significant increases in bioavailable active carbon fractions. Dissolved organic carbon (DOC) increased by 47.2%, humic acid (HA) by 38.6%, and fulvic acid (FA) by 29.4% (*P* < 0.05). These active carbon fractions are not directly introduced by coal gangue itself; rather, their significant elevation is driven by the microbial degradation and transformation of inert carbon components mediated by *B. aerius*. Furthermore, the Y33-2 treatment significantly increased soil moisture content compared to other groups. Enhanced water retention is critical for supporting plant physiological functions, particularly under moisture-limited conditions (40).

**TABLE 1:** Physicochemical properties of soil under different treatments

Treatment	Organic matter (g/kg)	DOC (mg/kg)	Humic acid (g/kg)	Humin (g/kg)	Fulvic acid (g/kg)	Moisture content (%)	Total nitrogen (g/kg)	Total phosphorus (g/kg)	Total potassium (g/kg)	Cation exchange capacity (cmol/kg)	pH
C-1	41	88	21	14	06	13	10	20	67	33	82
Y33-1	12	51	61	36	25	40	12	21	74	98	43
C-2	28	22	84	56	28	01	23	22	18	98	88
Y33-2	61	69	04	66	38	50	32	25	93	88	38

Analysis of essential plant nutrients revealed notable improvements in the Y33-2 treatment. Concentrations of total nitrogen (N), phosphorus (P), and potassium (K) were elevated to varying degrees. Most significantly, the Y33-2 group exhibited markedly higher levels of total phosphorus and total potassium compared to all other treatments. These elements are vital for plant development, yield, and stress tolerance (41). Collectively, these results demonstrate that the synergistic application of *B. aerius* and coal gangue enhances key soil quality parameters, including organic matter, moisture retention, and nutrient availability (particularly P and K), thereby creating favorable conditions for *Medicago sativa* growth.

### Impacts of coal gangue and *B. aerius* on soil enzyme activities

**Fig 2:**
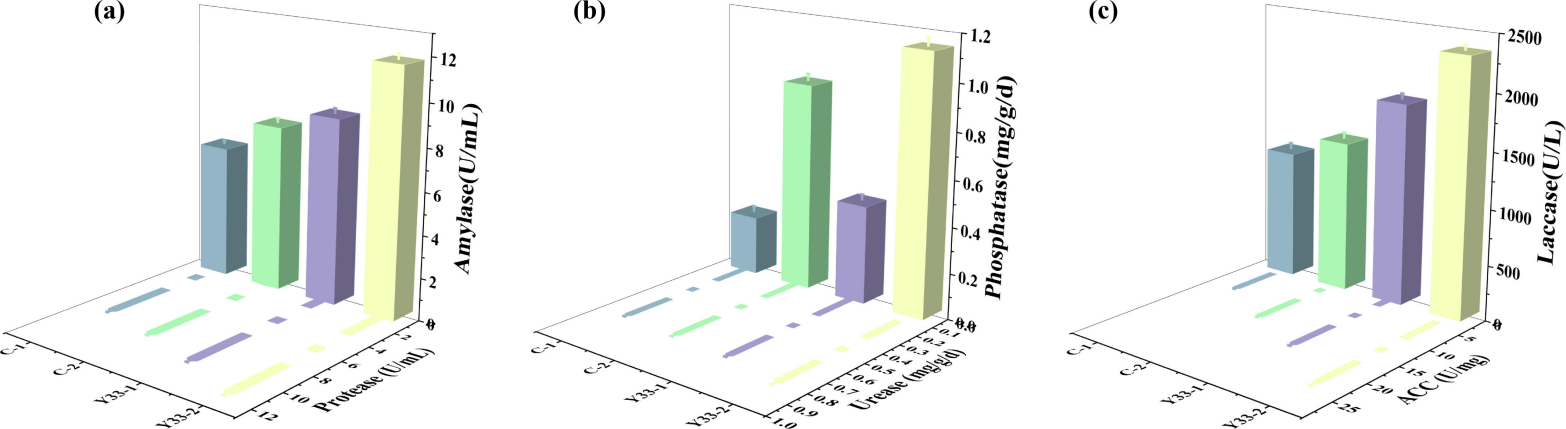
Soil enzyme activities across experimental treatments. (**a**) Amylase and protease activities, (**b**) urease and phosphatase activities, and (**c**) laccase and ACC deaminase activities.

Soil enzyme activities are critical catalysts for nutrient cycling, soil structural improvement, pollutant degradation, and plant development (42–45). As shown in Fig. 2, the combined coal gangue and *B. aerius* treatment (Y33-2) significantly enhanced key enzyme activities relative to the untreated control (C-1). Urease and protease activities increased by 15% and 44%, respectively.

Urease facilitates nitrogen cycling by converting urea into a nitrogen source accessible to plants, while protease contributes to nitrogen recycling by decomposing organic nitrogen sources. Both enzymes are essential for nitrogen absorption in plants (46–48). Activities of laccase and amylase increased by 99% and 84%, respectively. Laccase promotes organic matter decomposition and humification (49, 50), while amylase hydrolyzes starch to soluble sugars, influencing soil carbon dynamics (51). Most notably, phosphatase activity increased by 335% (4.35-fold) in the Y33-2 group compared to the untreated group. This enzyme is essential for mineralizing organic phosphorus, significantly enhancing phosphorus bioavailability, particularly crucial in phosphorus-limited soils (52).

### Plant growth hormones and ACC deaminase activity

**Fig 3:**
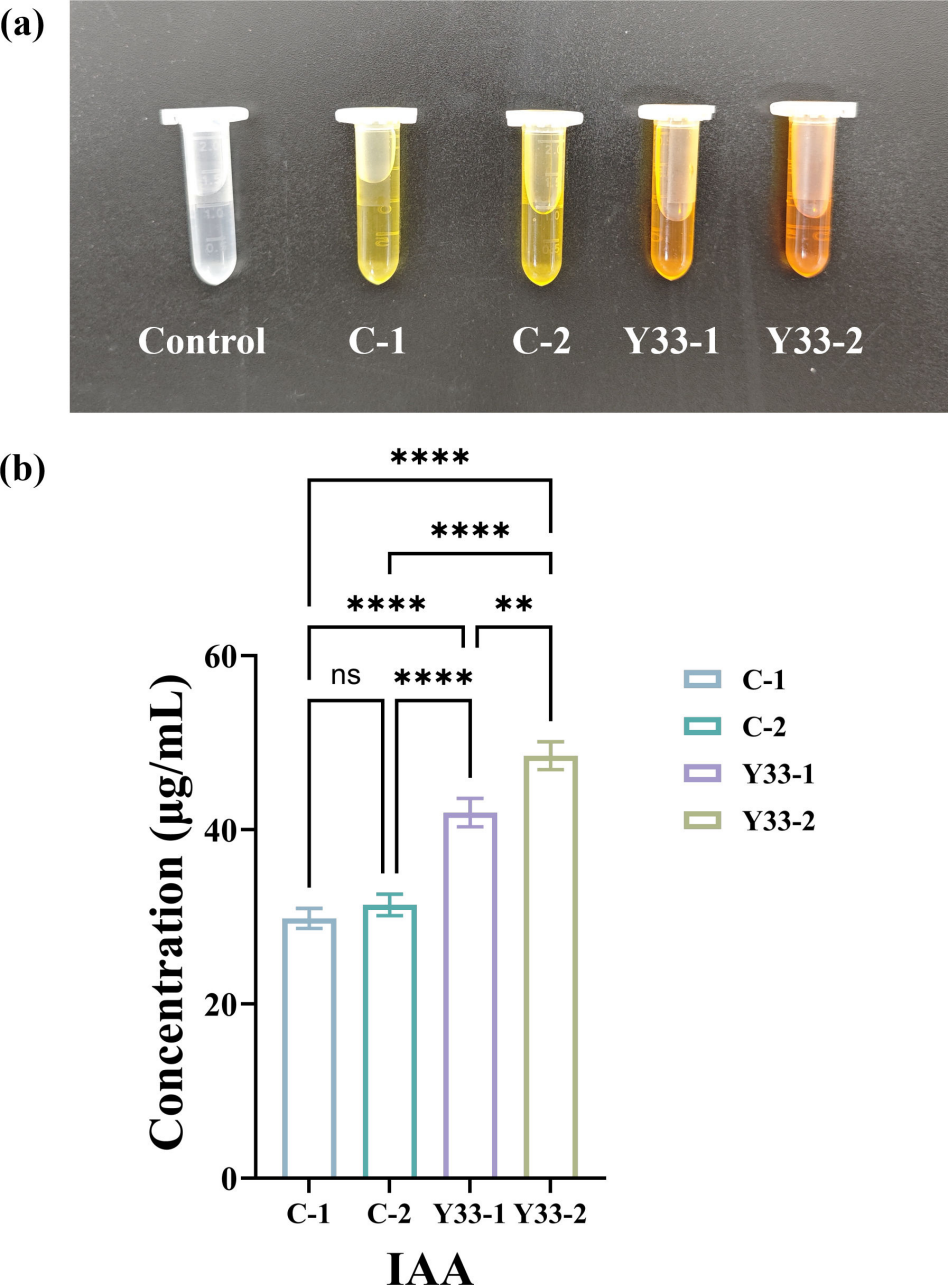
IAA activity in soils. (**a**) Colorimetric analysis of IAA activity across different experimental treatments and (**b**) quantification of IAA activity in different soil treatments. (****, *P* < 0.0001; **, *P* < 0.01).

IAA, a key plant auxin, regulates critical growth processes and stress responses (31). Bacteria enhance IAA levels either through direct synthesis or by stimulating endogenous production in plant roots (53, 54). *Bacillus spp*. are known to promote plant growth and stress tolerance via IAA secretion (55). As shown in Fig. 3, IAA was consistently detected throughout alfalfa growth. Bacterial inoculation significantly increased soil IAA content across experimental groups. Most notably, the combined coal gangue and *B. aerius* treatment (Y33-2) exhibited IAA levels 63% higher than the untreated control (C-1) and 55% higher than gangue alone (C-2) (*P* < 0.001).

Similarly, ACC deaminase activity increased dramatically in the Y33-2 treatment, reaching levels 152% higher (2.52-fold) than the C-1 control (Fig. 2c). This enzyme enhances plant growth by cleaving the ethylene precursor ACC, thereby reducing ethylene-mediated growth inhibition and promoting root development under stress (56, 57). These results indicate that bacteria have enhanced the activity of various biological enzymes in coal gangue soil, which collectively ensure soil nutrient supply and promote alfalfa growth. Collectively, these results demonstrate that *B. aerius* significantly enhances key growth-promoting activities, including IAA production and ACC deaminase expression in coal gangue-amended soil, contributing to improved nutrient availability and alfalfa growth.

### Soil microbial diversity analysis

**Fig 4:**
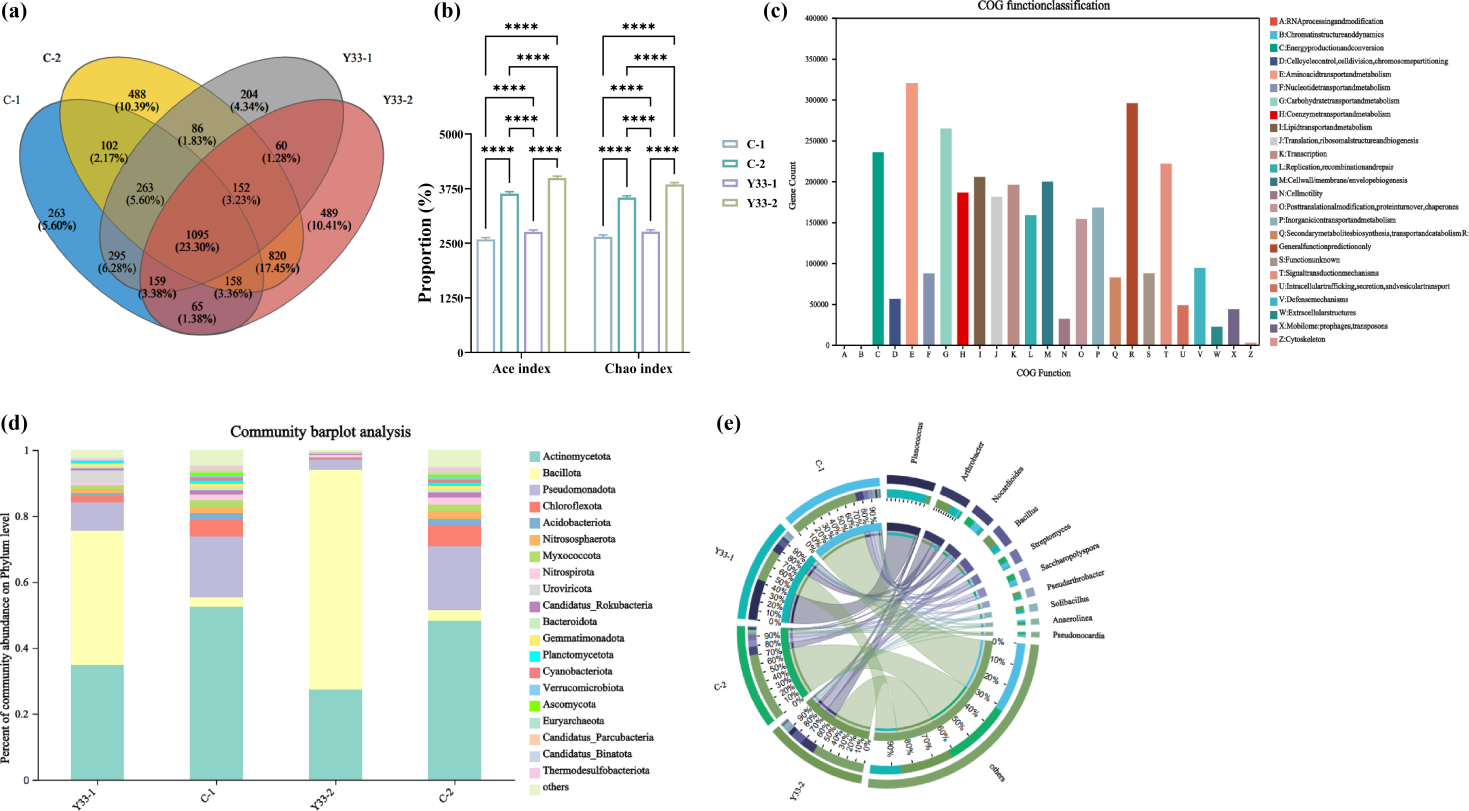
Microbial diversity across experimental soil treatments. (**a**) Venn diagram showing the overlap and unique OTUs in different soil treatments and (**b**) diversity indices. (**c**) Functional categorization of Clusters of Orthologous Groups (COG). Each bar represents the relative abundance of functional categories identified in the data set, with the categories grouped accordingly. (**d**) Soil bacterial community composition across experimental treatments at the genus level. (****, *P* < 0.0001). (**e**) Circos plot showing the distribution of dominant bacterial genera across treatments.

High-throughput sequencing generated 341,213 quality-filtered sequences (142,760,207 bases; average length 418 bp). Clustering at 97% similarity identified 5,186 bacterial operational taxonomic units (OTUs) across all treatments. The combined coal gangue and *B. aerius* treatment (Y33-2) exhibited the highest OTU richness (2,998 OTUs), representing an increase of 32.37% and 16.45% compared to the untreated control (C-1) and coal gangue-only treatment (C-2), respectively (Fig. 4a). A core microbiome of 1,209 OTUs (23.30% of total OTUs) was shared among all treatments. Sequencing coverage exceeded 98.65% for all treatments, confirming adequate capture of community structure. Bacterial inoculation significantly enhanced microbial richness indices. Both Ace and Chao indices, which are indicative of species richness, increased in coal gangue-amended soil following bacterial addition (Fig. 4b; also see Table S2 at https://doi.org/10.6084/m9.figshare.31852120). Further analysis revealed that the Ace and Chao indices of soil bacteria treated with Y33-1 were higher than those of C-1, while the indices for Y33-2-treated soil were higher than those of C-2. Notably, the Y33-2 treatment produced the most significant results, with the indices ranked as follows: Y33−2 > C-2 > Y33-1 > C-1. In contrast, Simpson and Shannon diversity indices showed no significant differences between control and inoculated treatments. This demonstrates that *B. aerius* primarily enhances microbial community richness rather than diversity in coal gangue-amended soil.

### Treatment-driven restructuring of soil bacterial community composition

High-throughput sequencing revealed significant restructuring of the soil bacterial community following *B. aerius* inoculation in coal gangue-amended soil (Y33-2 treatment). The 10 most abundant genera across treatments including *Planococcus, Arthrobacter, Nocardioides, Bacillus, Streptomyces, Saccharopolyspora, Pseudarthrobacter, Solibacillus, Anaerolinea,* and *Pseudonocardia* are visualized in Fig. 4d and e. Critically, the Y33-2 treatment exhibited dramatic enrichment of plant growth-promoting genera relative to unamended (C-1) and gangue-only (C-2) controls. *Bacillus* abundance increased by 15.52-fold in Y33-2 compared to controls. Similarly, *Arthrobacter* and *Pseudarthrobacter* increased by 7.85-fold and 11.21-fold, respectively. *Solibacillus*, which is undetectable in C-1, emerged in all inoculated treatments.

These shifts in the microbial community are consistent with the roles of the identified genera in promoting plant growth. *Bacillus* species are known for their ability to produce plant hormones, enhance disease resistance, and improve nutrient cycling in the soil (58–60). Similarly, *Arthrobacter* species contribute to the decomposition of organic matter and hormone production, which further supports plant health and growth (61). *Streptomyces* enhances biomass synthesis and exhibits antimicrobial activity (62). *Pseudarthrobacter* has been shown to inhibit pathogenic bacteria and promote plant growth through hormone synthesis (63), and *Solibacillus* functions through analogous mechanisms (64). Overall, these findings demonstrate that *B. aerius* inoculation restructures the soil microbiome by selectively enriching plant growth-promoting taxa, which likely contributes to the enhanced development of alfalfa observed in the Y33-2 treatment.

### Reprogramming of soil functional metagenomes

Metagenomic analysis identified several key plant growth-promoting genes within the soil microbiome. The *trpCDE* gene cluster, involved in IAA biosynthesis via tryptophan metabolism (65), and the *acdS* gene, encoding ACC deaminase (66), were both detected. ACC deaminase catalyzes the cleavage of ACC into ammonia and α-ketobutyrate, thereby reducing ethylene biosynthesis in plants and promoting growth (67). Clusters of Orthologous Groups (COG) functional categorization revealed that the most abundant functional category was amino acid transport and metabolism (Category E), followed by general function prediction (Category R) and carbohydrate transport and metabolism (Category G) (Fig. 4c). The predominance of metabolic functions suggests that microbial-mediated plant growth promotion primarily occurs through enhanced amino acid and carbohydrate metabolic pathways (68–70).

**Fig 5:**
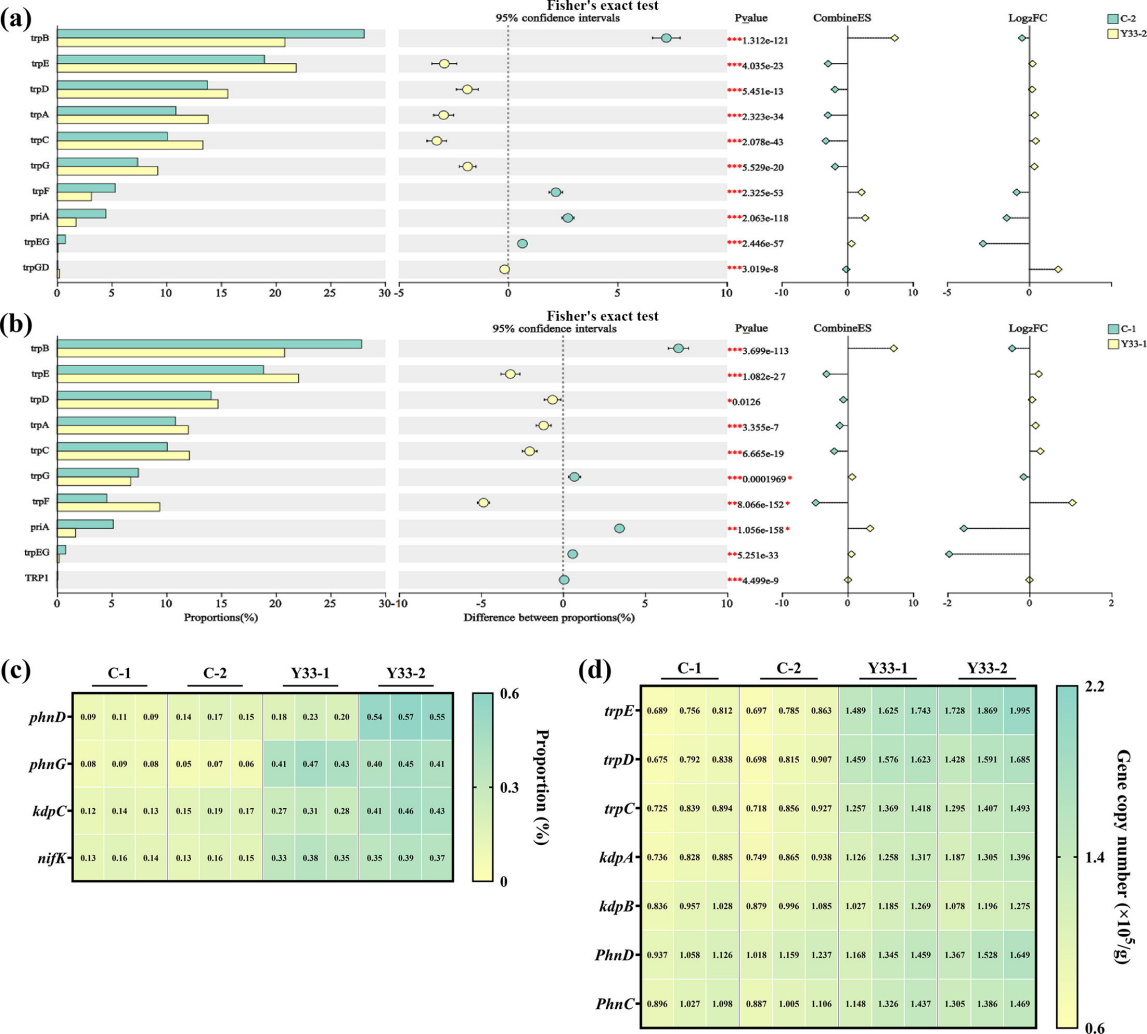
Comparative analysis of IAA-related gene abundance levels between different experimental groups. (**a**) Comparison between C-2 and Y33-2 groups and (**b**) comparison between C-1 and Y33-1 groups. Expression and transcription levels of key genes across different experimental groups. (**c**) Comparison of gene abundance related to phosphorus (*phnD*), potassium (*KdpC*), and nitrogen (*nifK*) metabolism pathways and (**d**) comparison of gene transcription levels associated with IAA synthesis (*trpCDE*), phosphorus (*phnDG*), potassium (*KdpC*), and ACC pathways. Error bars represent the standard deviation of the mean from replicates.

Supplementation with *B. aerius* significantly upregulated genes critical for plant growth promotion (Fig. 5a through c). The experimental group exhibited elevated expression of *trpE*, *trpD*, *trpC*, and *trpA*, genes essential for the IAA biosynthetic pathway. This upregulation enhances L-tryptophan metabolism, driving increased IAA production. Furthermore, the addition of *B. aerius* substantially enhanced gene expression in phosphorus, potassium, and nitrogen metabolic pathways. Specifically, *B. aerius* elevated the expression of the *phnD* and *phnG* genes, which are essential for soil phosphorus cycling. The *phnD* gene is involved in the degradation of organic phosphorus compounds, while *phnG* participates in the transformation of organic phosphorus sources (71). Activation of these genes significantly increased the availability of phosphorus in the soil, thereby enhancing nutrient supply and improving plant phosphorus uptake (72). Furthermore, *B. aerius* also stimulated the expression of the *KdpC* gene, which is essential for potassium ion transport. The *KdpC* gene regulates the active transport of potassium ions, maintaining intracellular potassium homeostasis (73). Additionally, the bacterium influenced the *nifK* gene, a key player in nitrogen fixation. The *nifK* gene encodes a subunit of nitrogenase, which facilitates the conversion of atmospheric nitrogen into ammonia nitrogen that plants can utilize (74). By promoting the expression of *nifK*, *B. aerius* enhanced nitrogen availability in the soil, which is particularly beneficial for plant growth in nitrogen-deficient soils (75).

Quantitative reverse transcription-PCR (qRT-PCR) analysis corroborated these findings (Fig. 5d), confirming that *B. aerius* enhances transcriptional activity of genes governing plant growth-promoting mechanisms. Collectively, these results demonstrate that *B. aerius* improves soil nutrient content while simultaneously promoting plant growth through phytohormone regulation and enhanced nutrient cycling.

## DISCUSSION

The treatment and resource utilization of coal gangue have gained significant attention due to growing environmental concerns (75). While previous studies have established that certain bacterial species can degrade harmful components within coal gangue, the present study advances this field by demonstrating that *Bacillus aerius* synergistically interacts with coal gangue to promote the growth of *Medicago sativa* through multiple complementary mechanisms. Building upon our previous finding that *B. aerius* facilitates coal gangue degradation (76), the current results show that this bacterial amendment, under optimized conditions, significantly improves alfalfa biomass and soil quality. This aligns with research emphasizing the necessity of microbial partners for unlocking the agronomic potential of mineral amendments (26). Specifically, *B. aerius* enhanced soil organic matter and humic acid content, consistent with reported *Bacillus*-mediated humification processes (77). Furthermore, the bacterium’s capacity to solubilize the mineral matrix of inherently nutrient-deficient coal gangue via organic acid secretion emerges as a critical mechanism for its resource utilization (78).

Mechanistically, *B. aerius* and coal gangue jointly enhanced key soil enzyme activities, including protease, laccase, urease, phosphatase, and amylase, as well as plant growth regulators such as IAA and ACC deaminase. Tomás et al. have demonstrated that plant-growth-promoting rhizobacteria (PGPR) significantly influence plant growth by secreting enzymes that enhance nutrient availability in the soil (79). Collectively, these enzymes facilitate the decomposition of organic matter and the release of essential minerals, thereby promoting nutrient cycling and providing robust nutritional support for alfalfa growth. Notably, the elevated ACC deaminase and IAA levels confirm that *B. aerius* directly stimulates alfalfa growth via phytohormone regulation, complementing the indirect growth-promoting effect of soil nutrient improvement.

**Fig 6:**
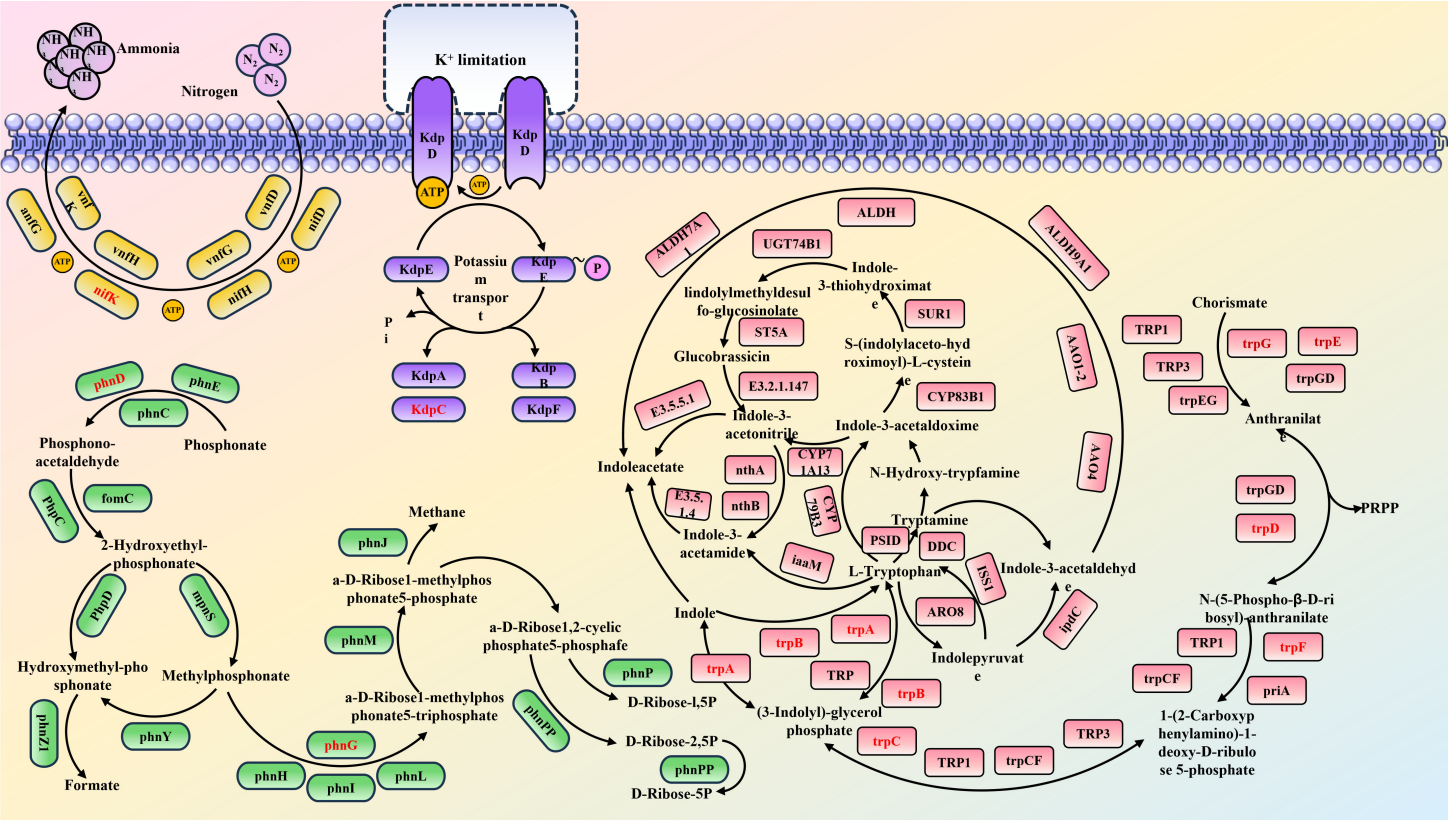
Schematic representation of metabolic pathways for phosphorus, potassium, and nitrogen cycling, and IAA biosynthesis. Genes with increased expression are highlighted in red.

Microbial community analysis revealed that *B. aerius* significantly increased the richness (OTU number) and restructured the microbial community composition, enriching beneficial genera such as *Bacillus*, *Arthrobacter*, and *Pseudarthrobacter*. This shift creates a more favorable rhizosphere microenvironment for alfalfa growth, as these enriched taxa are widely recognized to enhance nutrient solubilization, phytohormone secretion, and pathogen inhibition, consistent with previous reports that *Bacillus spp*. optimize rhizosphere microbial communities to support plant growth (57). Furthermore, the study revealed upregulation of key genes involved in plant growth and soil nutrient cycling, such as *trpEDAC*, *phnDG*, *KdpC*, and *nifK* (Fig. 6). Metagenomic and transcriptomic analyses indicated that the expression of these genes was significantly increased, suggesting that the associated metabolic pathways for phosphorus, potassium, and nitrogen cycling are activated. By regulating these genes, *B. aerius* influences soil enzyme activity and growth hormone levels, facilitating the efficient cycling of soil nutrients. This finding provides valuable insights into the mechanisms that underpin the beneficial effects of coal gangue as a mineral soil fertilizer, offering a promising solution for its environmentally sustainable utilization.

In summary, this study provides a comprehensive understanding of the growth-promoting effects of the combined application of coal gangue and *B. aerius* on alfalfa, along with the biological mechanisms associated with this process. *B. aerius* plays a crucial regulatory role by influencing gene expression, enzymatic activity, and growth hormone levels, which not only enhance nutrient cycling within the soil but also propose innovative strategies for the resource utilization of coal gangue and environmental sustainability. This study establishes an effective and eco-friendly strategy for coal gangue utilization through a *B. aerius*-coal gangue synergistic system, demonstrating clear application potential for the remediation of degraded sandy soil. However, two core limitations of this work must be acknowledged. First, as all experiments were conducted in indoor pot trials, the long-term stability and field-scale performance of this synergistic system require further validation. Second, this investigation focused on the growth-promoting effects and mechanisms of the system but paid little attention to soil-available heavy metal concentrations or leaching toxicity at the end of the pot experiment. This limitation is particularly relevant given that the presence of heavy metals and organic pollutants in coal gangue remains a concern for ecological systems (80, 81). Research has confirmed that coal gangue contains trace levels of naturally occurring radioactive substances, including uranium and thorium, as well as their radioactive decay byproducts (82). Furthermore, coal gangue has been found to contain toxic compounds such as polycyclic aromatic hydrocarbons, which may contaminate soil (83). Notably, several lines of evidence from this study mitigate these concerns. The pH of coal gangue-amended soil decreased from 8.82 (strongly alkaline) to 7.38 (near-neutral), indicating that acidic conditions (pH < 7) capable of driving heavy metal activation did not develop. Classic soil environmental chemistry theory confirms that near-neutral pH corresponds to the lowest bioavailability for most heavy metals, theoretically mitigating the risk of heavy metal migration. Furthermore, end-of-experiment total heavy metal detection results verified that *B. aerius* inoculation significantly reduced the accumulation of typical heavy metals in coal gangue-amended soil, particularly for Cr and Hg (see Table S5 at https://doi.org/10.6084/m9.figshare.31852120). These data provide direct experimental support for the environmental safety of this amendment strategy. Future field studies will include the detection of available heavy metal fractions and leaching toxicity to comprehensively assess long-term environmental risk.

### Conclusions

This study provides a comprehensive analysis of the synergistic effects of coal gangue and *B. aerius* on plant growth and soil health. The co-application of coal gangue and *B. aerius* to sandy soil led to significant improvements in alfalfa growth, with the germination rate, shoot height, root length, and fresh biomass increasing by 1.18-fold, 1.91-fold, 2.56-fold, and 2.06-fold, respectively. These results highlight the substantial growth-promoting effects of this combination. Additionally, the concentrations of key soil nutrients, including nitrogen, phosphorus, and potassium, were markedly elevated, suggesting an enhancement in soil fertility. Beyond nutrient enrichment, the bacterial treatment also significantly increased the activity of soil enzymes and growth-promoting factors. Notable increases in the activities of IAA, ACC deaminase, urease, and phosphatase underscore the potential of *B. aerius* to modulate soil biochemical properties in a way that fosters plant development. Furthermore, bacterial inoculation resulted in changes to the soil microbial community structure, including increased species richness and a higher abundance of beneficial genera, such as *Bacillus*, *Arthrobacter*, and *Pseudarthrobacter*. At the molecular level, the upregulation of genes associated with plant growth, such as *acdS*, *KdpC*, *nifK,* and *phnG*, supports the involvement of multiple mechanisms, such as enhanced soil nutrient content, hormone regulation, and nutrient cycling, that contribute to the observed growth effects. Together, these findings deepen our understanding of the interactive mechanisms between *B. aerius* and coal gangue, providing a scientific foundation for their application in sustainable soil amendment and ecological restoration strategies.

## Data Availability

The metagenomic sequence data from this study were deposited in the Sequence Read Archive (SRA) of the National Center for Biotechnology Information (NCBI) under accession number PRJNA1363089.
